# Supplementary material for: Validation of a Food Knowledge Questionnaire on Tanzanian Women of Childbearing Age
Source: Nutrients. 2022 Feb 7;14(3):691. doi: 10.3390/nu14030691 (PMC8840700; doi:10.3390/nu14030691)
Supplement: Supplementary file 1 [file nutrients-14-00691-s001.zip › nutrients-1573148-supplementary.pdf]

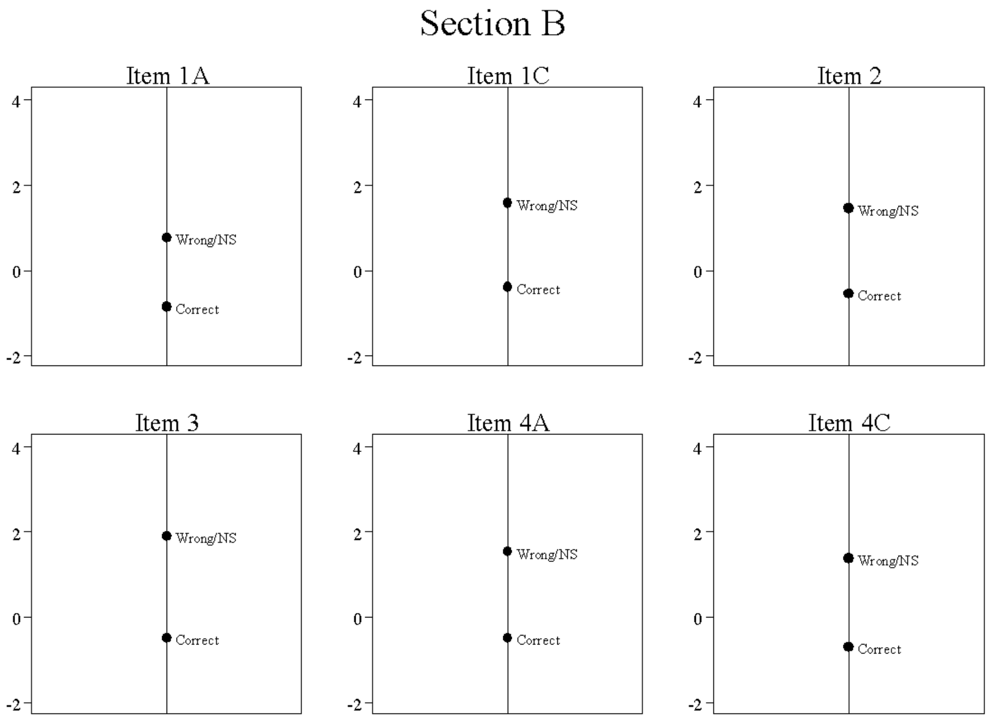

Figure S1. MCA Projection Plot for Section B. Coordinates in standard normalisation.

Item identifiers as in Table N3.

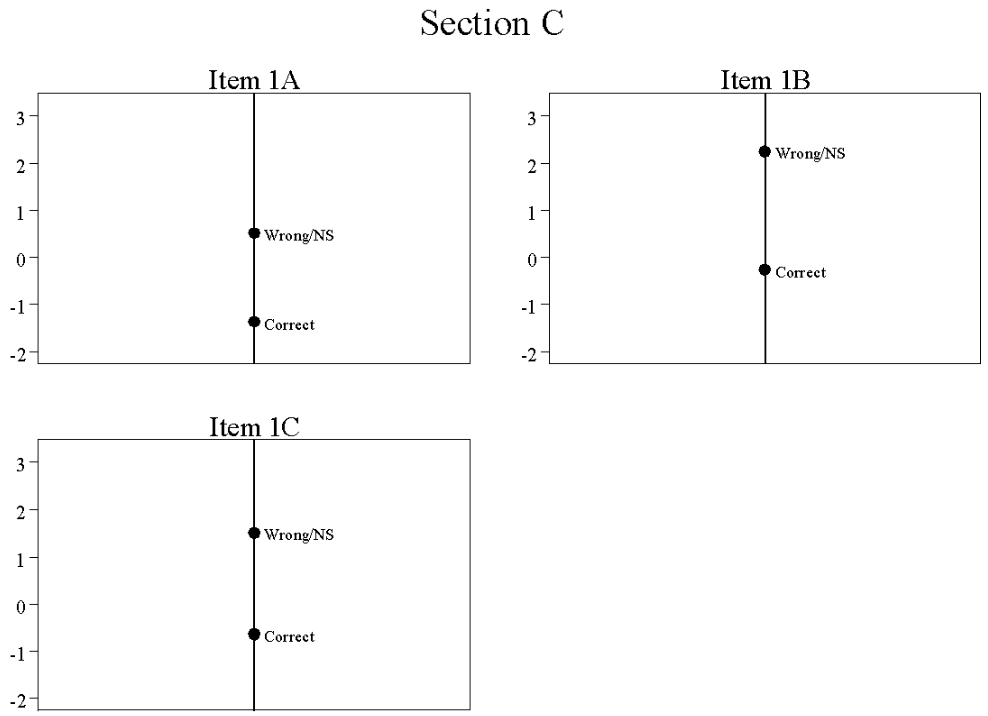

Figure S2. MCA Projection Plot for Section C. Coordinates in standard normalisation.

Item identifiers as in Table N3.

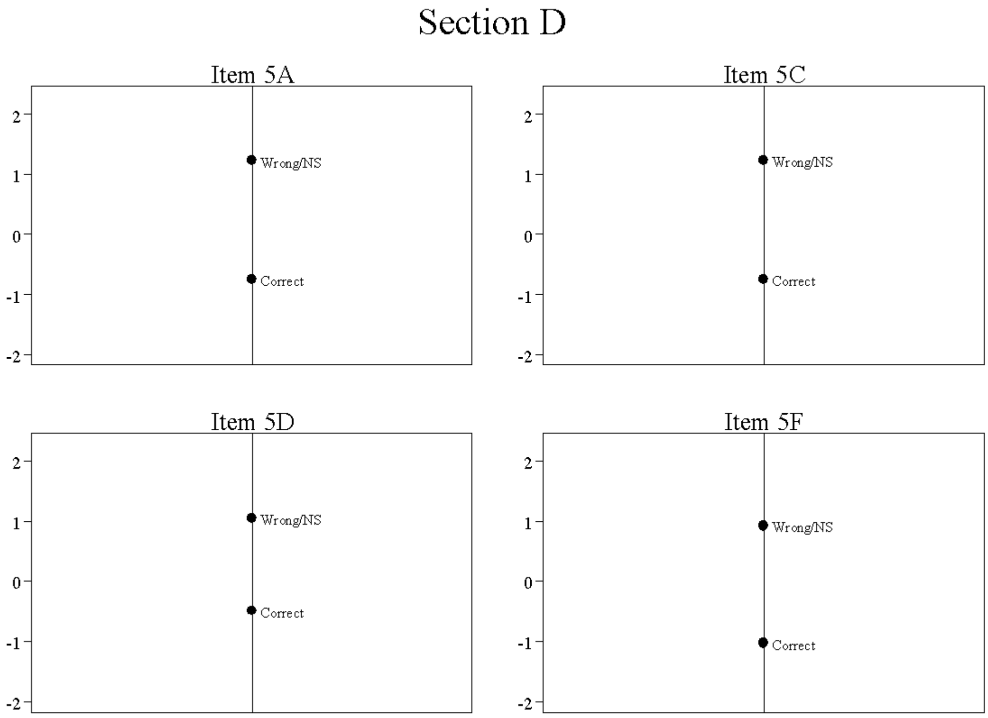

Figure S3. MCA Projection Plot for Section D. Coordinates in standard normalisation.

Item identifiers as in Table N3.

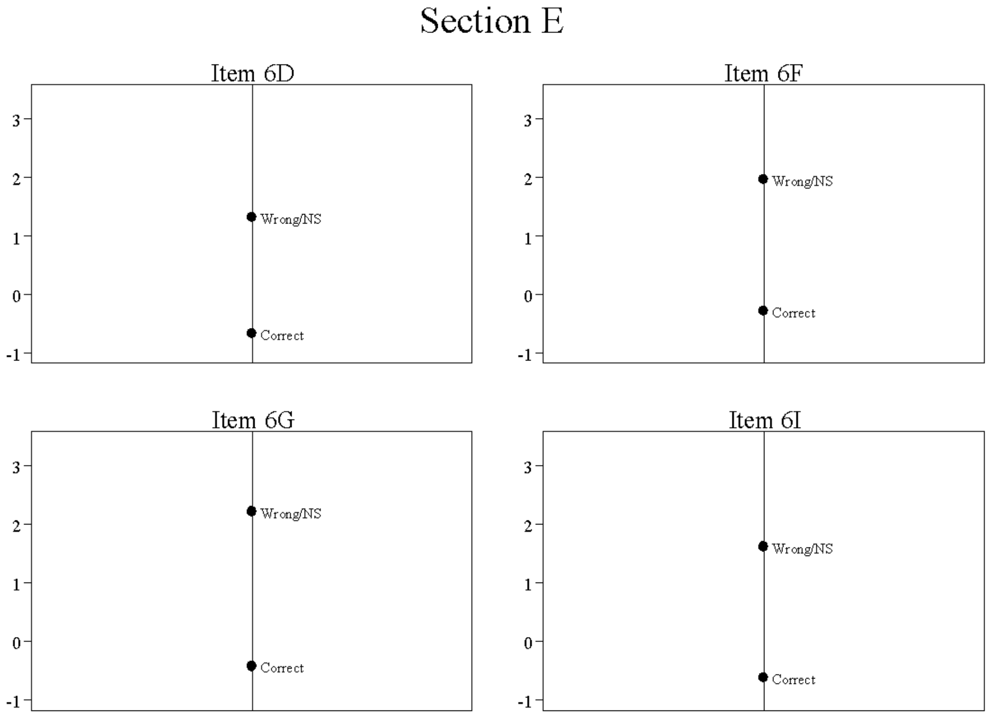

Figure S4. MCA Projection Plot for Section E. Coordinates in standard normalisation.

Item identifiers as in Table N3.

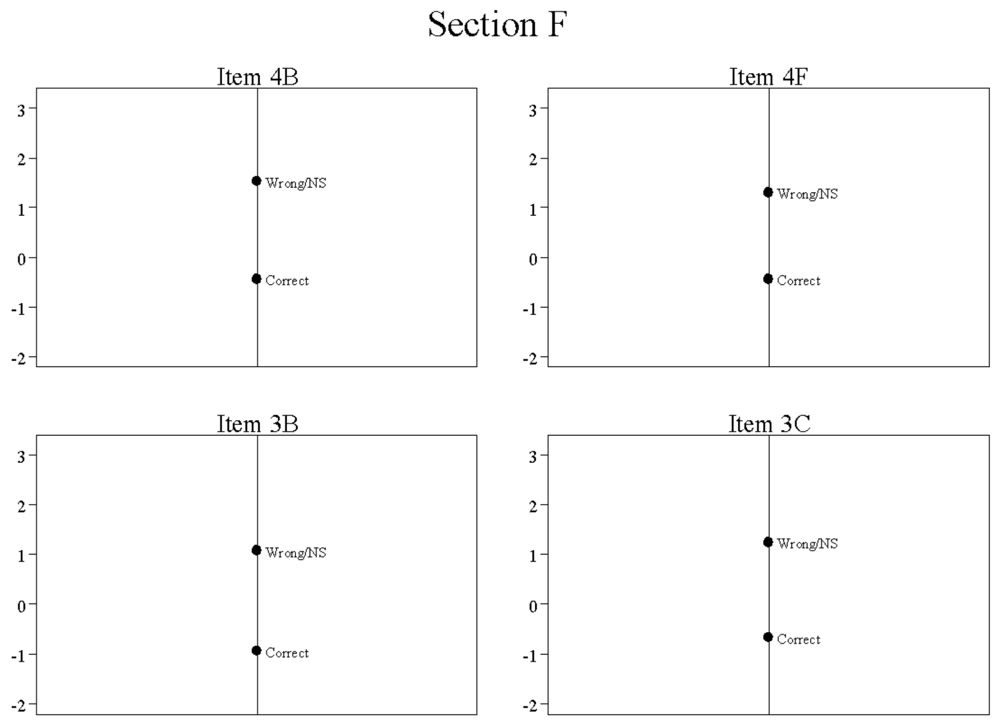

Figure S5. MCA Projection Plot for Section F. Coordinates in standard normalisation.

Item identifiers as in Table N3.

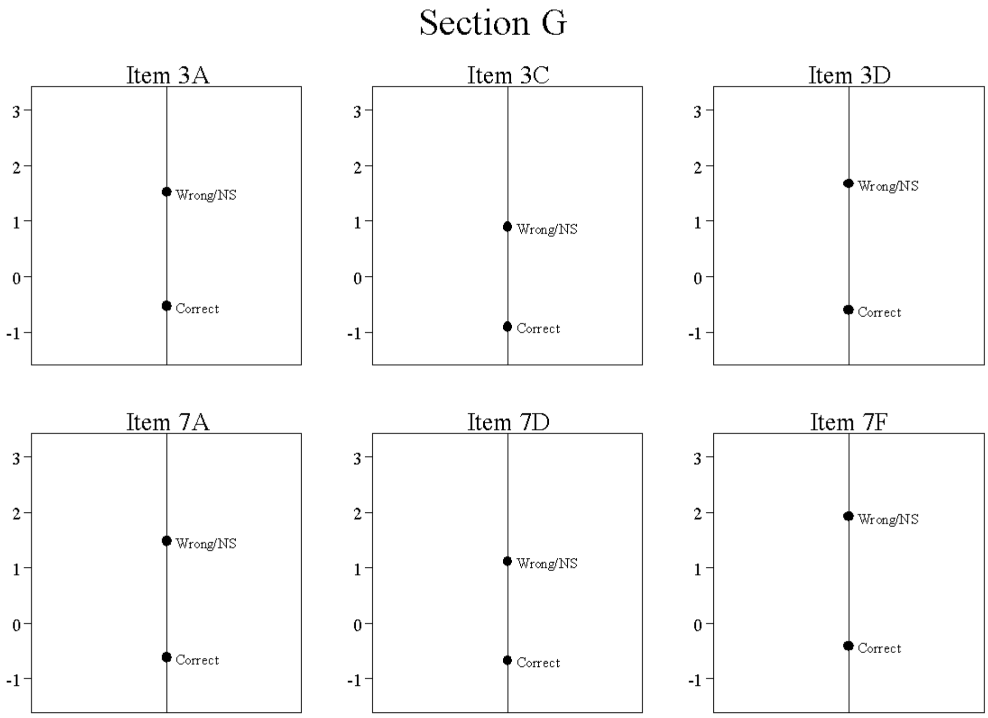

Figure S6. MCA Projection Plot for Section G. Coordinates in standard normalisation.

Item identifiers as in Table N3.

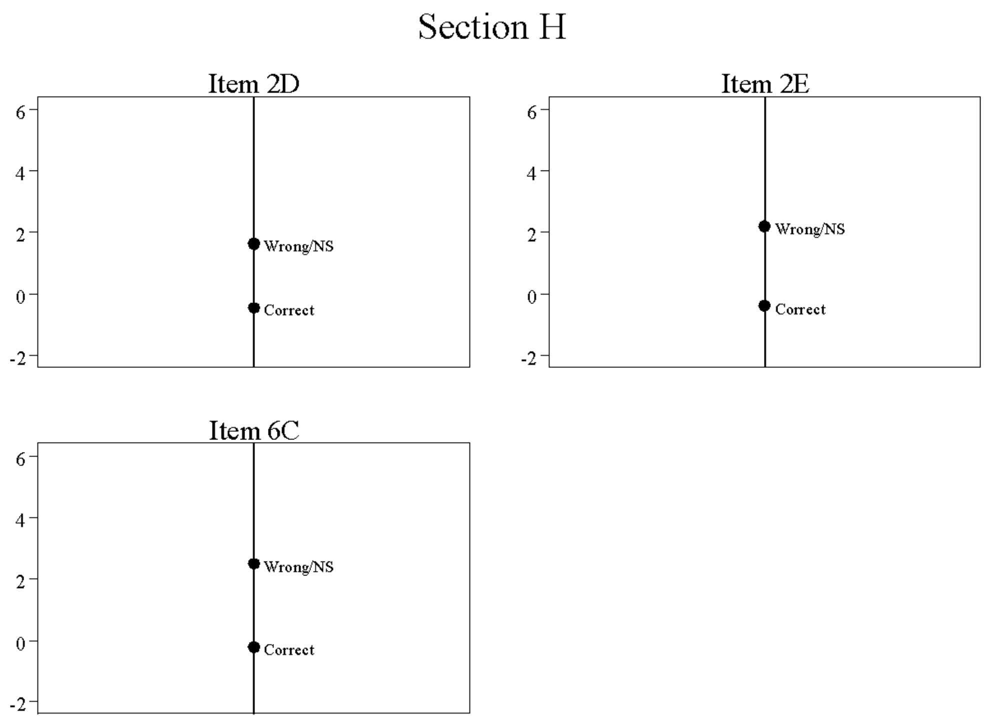

Figure S7. MCA Projection Plot for Section H. Coordinates in standard normalisation.

Item identifiers as in Table N3.

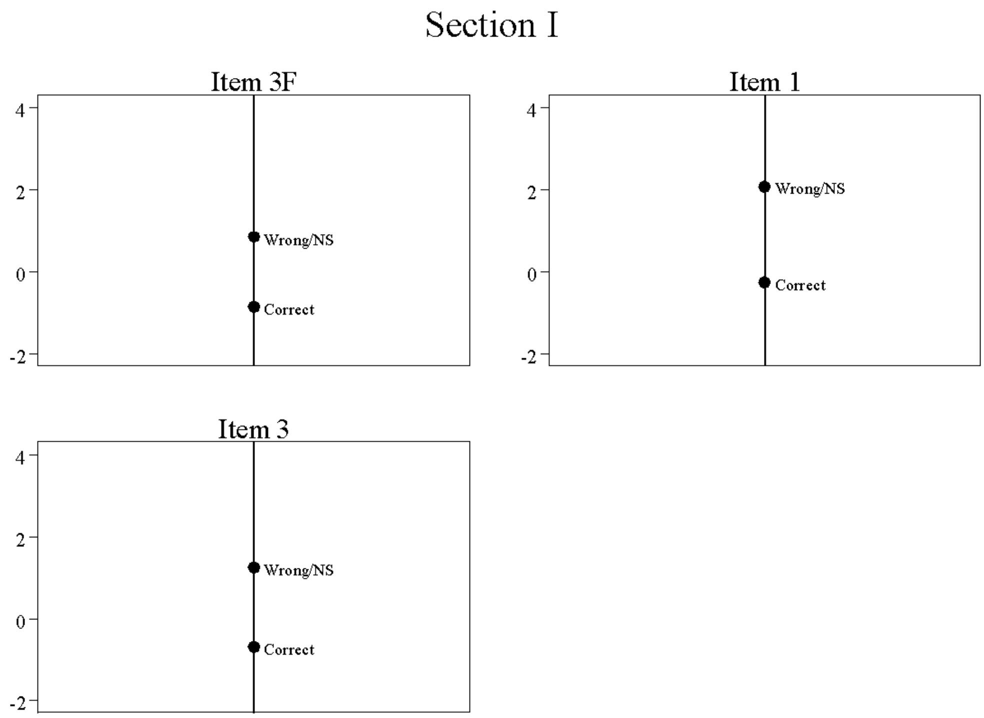

Figure S8. MCA Projection Plot for Section I. Coordinates in standard normalisation.

Item identifiers as in Table N3.

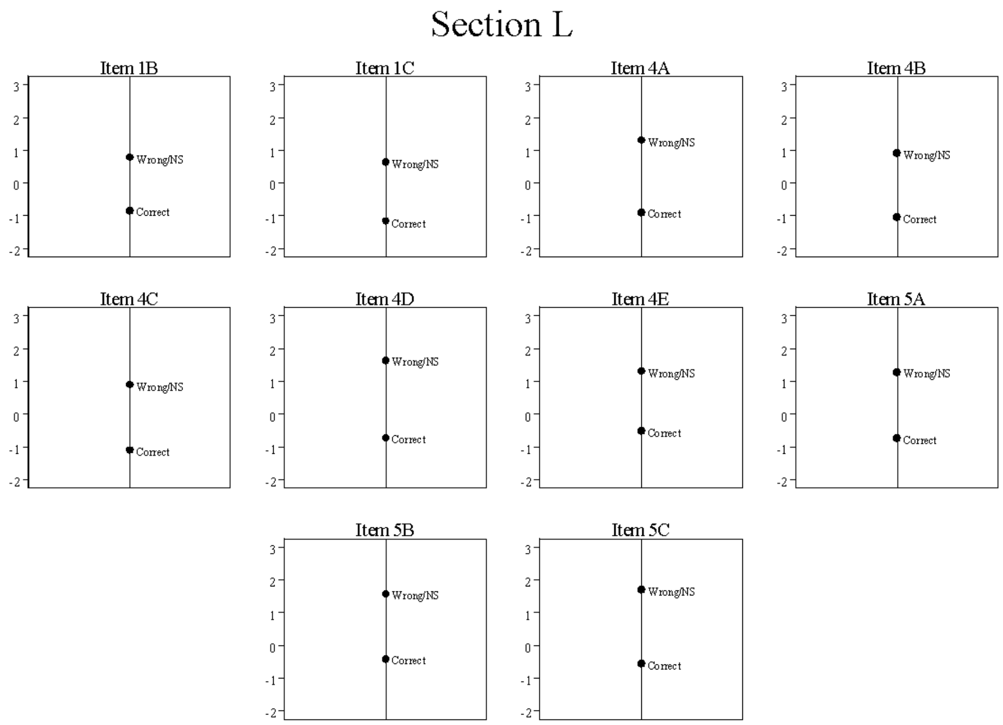

Figure S9. MCA Projection Plot for Section L. Coordinates in standard normalisation.

Item identifiers as in Table N3.

Table S1. Factors' scores and schooling level.

Section	Schooling level			Kruskal Wallis' test		Post-hoc tests§		
	*None**	*Primary/ Second.**	*Higher**	*Test*	*p-value*	*None vs. Prim/Sec*	*None vs. Higher*	*Prim/Sec vs. Higher*
B	2 (2)	4 (2)	5 (1)	59.71	<0.001	X	X	X
C	2 (1)	2 (1)	2 (2)	7.47	0.024		X	
D	1 (2)	2 (3)	4 (1)	73.25	<0.001	X	X	X
E	2 (2)	4 (2)	4 (1)	35.39	<0.001	X	X	X
F	2 (2)	3 (2)	3 (2)	15.26	<0.001	X	X	
G	3 (3)	4 (3)	6 (2)	69.84	<0.001	X	X	X
H	2 (2)	3 (1)	3 (1)	17.45	<0.001	X	X	
I	1 (1)	2 (1)	2 (1)	24.95	<0.001	X	X	
L	5 (7)	6 (5)	7 (4)	20.49	<0.001		X	X

*Median (IQR). §Bonferroni-adjusted significance. The only significance is reported as an "X" symbol.

Table S2. Sections' scores and nutrition-related qualifications.

Section	Nutrition-related qualification		Mann-Whitney' test	
	*No**	*Yes**	**Test**	**p-value**
B	5 (2)	3 (2)	2.91	0.004
C	2 (1)	1.5 (1)	1.86	0.064
D	3 (3)	2 (2)	1.51	0.132
E	4 (2)	3 (2)	0.73	0.466
F	3 (2)	3 (2)	0.22	0.827
G	5 (3)	4 (2)	1.01	0.313
H	3 (1)	3 (0.5)	-0.61	0.543
I	2 (2)	2 (1)	-1.39	0.163
L	6 (4)	4 (7.5)	0.75	0.454

*Median (IQR).

ANNEX A

Tanzanian Food Knowledge Questionnaire

**Nutrition survey**
This is a survey, NOT a test.<br>If you do not know the answer, reply "not sure" rather than guessing.

Consent form (Signed separately)

- o Yes
- o No

Name of Enumerator: ____________________________

Name of respondent: ____________________________

Date of administration: ___________________________

ID (reference code of the sample) | | | |

Coordinate GPS: __________________________________

SECTION A. SOCIO-DEMOGRAPHICS INFORMATION

1. What is your date of birth?

2. Where were you born? (Please specify)
Region……………………………………………………….
District……………………………………………….……….
Village/ Area……………………………………………..
Tribe…………………………………………………….……….

3. Where are you living now? (Please specify)
City/ Region…………………………………………………
District…………………………………………………………
Village/ Area………………………………………………

4. Which is your religion? …………………………….

5. How many alive children do you have? ………………………………………………

6. Do you have any child under 18 years living with you?

Yes	
No	

7. Which is the highest level of education you have completed?

A. No school education	
B. Primary school	
C. O Level secondary school	
D. A level (High school)	
E. Technical or tertiary certificate	
F. Diploma	
G. Degree	
H. Post-graduate degree	

8. Do you live with someone who has any nutrition-related qualification or who is currently a nutrition student?

Yes	
No	

If “Yes”, please specify the qualification…………………………………………………

9. Are you:

A. Single	
B. In a relationship	
C. Married	
D. Widow	
E. Divorced	

10. Do you have a disabled at the household level?

Yes	
No	

If “Yes”, please specify which kind of disability
………………………………………………………………………………………..

11. Do you have a job?

Yes	
No	

If “Yes”, please specify what is your job
………………………………………………………………………………………..

12. Who control the food expenditure?

A. Father	
B. Mother	
C. Children	
D. Other	

If “Other”, please specify
………………………………………………………………………………………..

13. How much money do you spend on food shopping per week?
TSH……………………………………………………………………………….

14. How much money do you earn per month? TSH ………………………………………………

15. How much money do your family totally earn per month? TSH ………………………….

16. Are you following any specific nutrition diet elaborated by a nutrition expert or by a healthcare specialist?

Yes	
No	

If “Yes”, please specify the reason…………………………………….
If “Yes”, please specify who elaborated the diet/therapy.

17. Where do you get information about nutrition?

A. Radio	
B. Health personnel (doctor, clinical attendants, nurse)	
C. Fliers	
D. Television	
E. Social media	
F. Religious gathering	
G. Family members	
H. Schools	
I. Markets	

SECTION B. THIS SECTION IS ABOUT WHAT ADVICE YOU THINK THE "HEALTH EXPERTS" ARE GIVING AT THE COMMUNITY TO MAINTAIN A GOOD HEALTH STATUS

1. Do "health experts" recommend eating a big quantity of the foods listed below to be in a good health status? (*tick one box per food)*

	AGREE	DISAGREE	NOT SURE
A. Starchy foods (processed)			
B. Foods high in fiber or roughage			

2. How many portions of fruit the "health experts" recommend consuming per day?
Choose only one of the options listed below.
(*One serving of fruit could be for example one apple or one orange*)

A. 0	
B. 2 servings	
C. 5 servings	
D. More than 5	
E. Not sure	

3. How many portions of vegetables the "health experts" recommend consuming per day?
Choose only one of the options listed below.
(*One serving of vegetable could be for example 1/2 plate of tomatoes/ carrots exc...)*

A. 0	
B. 2 servings	
C. 5 servings	
D. More than 5	
E. Not sure	

4. To maintain a good health status…do “health experts” recommend reducing the sources of fats listed below? (*tick one box per food)*

	AGREE	DISAGREE	NOT SURE
A. Pork / Beef			
B. Butter/ Margarine (Ghee)			

SECTION C. FOOD COMPOSITION: THE “HEALTH EXPERTS” ARE INTERESTED IN KNOWING WHETHER PEOPLE ARE AWARE OF SUGAR CONTENT IN FOOD

1. Do you think the foods listed below contain a lot of sugar? (*tick one box per food*)

	AGREE	DISAGREE	NOT SURE
A. Bananas			
B. Stiff porridge			
C. Plantain			

SECTION D. FOOD COMPOSITION: THE “HEALTH EXPERTS” ARE INTERESTED IN KNOWING WHETHER PEOPLE ARE AWARE OF PROTEIN CONTENT IN FOOD

1. Do you think the foods listed below are high in protein? (*tick one box per food*)

	AGREE	DISAGREE	NOT SURE
A. Mango			
B. Stiff porridge			
C. Fermented milk/ Yogurt			
D. Groundnuts			

SECTION E. FOOD COMPOSITION: THE “HEALTH EXPERTS” ARE INTERESTED IN KNOWING WHETHER PEOPLE ARE AWARE OF FIBER CONTENT IN FOOD

1. Do you think the foods listed below are high in fiber/roughage? (*tick one box per food*)

	AGREE	DISAGREE	NOT SURE
A. Beef			
B. Groundnuts			
C. Fish			
D. Chicken			

SECTION F. HEALTHY EXPERTS VERIFY THE KNOWLEDGE ABOUT SODIUM INTAKE/ SODIUM CONTENT IN FOOD AND HEALTH CONDITION

1. The following diseases or health problems are related to high sodium or salt intake:

	AGREE	DISAGREE	NOT SURE
A. DEFICIT VITAMIND D			
B. UNDERWEIGHT			

2. Do you think the products listed below are high in salt? (*tick one box per food)*

	AGREE	DISAGREE	NOT SURE
A. Chapati			
B. Groundnut paste			

SECTION G. THE "HEALTH EXPERTS" ARE INTERESTED IN KNOWING WHETHER PEOPLE ARE AWARE ABOUT FOOD CLASSIFICATION

1. Do you think "health experts" classify the foods listed below as starchy food?
(*tick-one box per food)*

	AGREE	DISAGREE	NOT SURE
A. Milk			
B. Beans			
C. Fish			

2. Do "health experts" consider the foods listed below as healthy alternatives to beef?
(*tick one box per food)*

	AGREE	DISAGREE	NOT SURE
A. Sweet potatoes			
B. Groundnuts			
C. Bread			

SECTION H. THE "HEALTH EXPERTS" ARE INTERESTED IN KNOWING WHETHER PEOPLE ARE AWARE OF FOOD NUTRIENT CONTENT

1. Do you think the foods listed below are high in fats? *(tick one box per food)*

	AGREE	DISAGREE	NOT SURE
A. Honey			
B. Eggs			

2. Do you think the foods listed below are high in fiber/roughage? (*tick one box per food*)

	AGREE	DISAGREE	NOT SURE
A. Eggs			

SECTION I. THE "HEALTH EXPERTS" WOULD UNSERSTAND IF PEOPLE ARE AWARE ABOUT THE CORRECT FOOD CHOICE WITH RESPECT TO THEIR NUTRITIONAL CHARACTERISTICS

1. Brown sugar is healthier than honey. (*tick one*)

AGREE	
DISAGREE	
NOT SURE	

2. White bread contains more vitamins and minerals than brown bread. (*tick one)*

AGREE	
DISAGREE	
NOT SURE	

3. Do you think "health experts" classify the foods listed below as starchy food? (*tick-one box per food)*

	AGREE	DISAGREE	NOT SURE
A.Sugar			

SECTION L. THIS SECTION IS ABOUT THE HEALTH PROBLEMS OR DISEASES ASSOCIATED WITH NUTRITION

1. Adopting the habit listed below help to reduce the chances of getting certain kinds of cancer: *(tick one box per food)*

	AGREE	DISAGREE	NOT SURE
A. Eating more fiber or roughage			

B. Eating less sugar			
C. Eating less salt			
D. Eating more fruits and vegetables			
E. Eating less preservatives/ additives			

2. Adopting the habit listed below help to prevent heart disease: *(tick one box per food)*

	AGREE	DISAGREE	NOT SURE
A. Eating more fiber or roughage			
B. Eating less salt			
C. Eating more fruits and vegetables			
